# RAGE Plays a Role in LPS-Induced NF-κB Activation and Endothelial Hyperpermeability

**DOI:** 10.3390/s17040722

**Published:** 2017-03-30

**Authors:** Liqun Wang, Jie Wu, Xiaohua Guo, Xuliang Huang, Qiaobing Huang

**Affiliations:** 1 Drug Discovery Research Center, Southwest Medical University, 319 Zhongshan Road, Luzhou 646000, China; liqunwang@swmu.edu.cn; 2 First Clinical College of Medicine, Southern Medical University, Guangzhou 510515, China; wujie11@smu.edu.cn; 3 Department of Pathophysiology, Key Lab for Shock and Microcirculation Research, Southern Medical University, Guangzhou 510515, China; lanblue@smu.edu.cn (X.G.); shock@smu.edu.cn (X.H.)

**Keywords:** LPS, RAGE, NF-κB, endothelial hyperpermeability

## Abstract

Endothelial functional dysregulation and barrier disruption contribute to the initiation and development of sepsis. The receptor for advanced glycation end products (RAGE) has been demonstrated to be involved in the pathogenesis of sepsis. The present study aimed to investigate the role of RAGE in lipopolysaccharide (LPS)-induced nuclear factor-κB (NF-κB) activation in endothelial cells and the consequent endothelial hyperpermeability. LPS-induced upregulation of RAGE protein expression in human umbilical vein endothelial cells (HUVECs) was detected by western blotting. Activation of NF-κB was revealed using western blotting and immunofluorescent staining. LPS-elicited endothelial hyperpermeability was explored by transendothelial electrical resistance (TER) assay and endothelial monolayer permeability assay. The blocking antibody specific to RAGE was used to confirm the role of RAGE in LPS-mediated NF-κB activation and endothelial barrier disruption. We found that LPS upregulated the protein expression of RAGE in a dose- and time-dependent manner in HUVECs. Moreover, LPS triggered a significant phosphorylation and degradation of IκBα, as well as NF-κB p65 nuclear translocation. Moreover, we observed a significant increase in endothelial permeability after LPS treatment. However, the RAGE blocking antibody attenuated LPS-evoked NF-κB activation and endothelial hyperpermeability. Our results suggest that RAGE plays an important role in LPS-induced NF-κB activation and endothelial barrier dysfunction.

## 1. Introduction

Sepsis remains an important cause of morbidity and mortality worldwide, especially in non-cardiac intensive care units. There has been no approved therapy for improving the status of septic patients to date. Recently, several Phase 3 clinical trials for sepsis therapy still exhibited disappointing outcomes [1,2]. Lipopolysaccharide (LPS), a major component of the cell wall of Gram-negative bacteria, is believed to be responsible for initiating exaggerated inflammatory cascades leading to sepsis.

Endothelial cells line the intima of blood vessels, being the first cells exposed to various pathogens in bloodstream. Vascular endothelium forms a vast network which dynamically regulates inflammation and maintains vascular integrity. Dysregulation of endothelial inflammatory pathways is believed to promote sepsis-related organ failure. Importantly, nuclear factor-κB (NF-κB) is the transcriptional factor of many inflammatory molecules, which are vital regulators of multiple organ failure in sepsis. The activation of NF-κB is associated with higher mortality in clinical sepsis [3]. NF-κB exists as heterodimer of proteins from the Rel family, including p50, p52, p65 (RelA), RelB, and c-Rel. The predominant form of NF-κB heterodimer consists of p50 and p65 subunits. The heterodimer resides in its resting state by associating with inhibitory proteins of IκB family. Once being activated by various pro-inflammatory and stress stimuli, IκBα undergoes phosphorylation, ubiquitination, and subsequent degradation. As a result, NF-κB heterodimer is released and further translocates to the nucleus. Then, the active form of NF-κB binds to decameric DNA sequence and activates the transcription of its target genes [4,5].

Endothelial hyperpermeability is caused by diffused endothelial injury and disruption of barrier function plays an important role in both early stage and development of sepsis [6]. Researchers have begun to explore the critical role of microvascular barrier as a therapeutic target in sepsis [7,8]. Our previous studies showed that LPS can induce F-actin rearrangement and endothelial hyperpermeability via p38 MAPK signaling pathway [9]. However, the precise mechanisms of LPS-increased endothelial permeability remain poorly understood. It is important to clarify the mechanisms of NF-κB activation and vascular barrier disruption induced by LPS, which may provide potential targets in pharmacologic treatment for sepsis.

Receptor of advanced glycation end products (RAGE), a member of the immunoglobulin super family, was first identified as the receptor for advanced glycation end products (AGEs) [10]. RAGE also interacted with a variety of other ligands, including S100A8/A9, high mobility group box1 (HMGB1), and LPS [11,12]. Previous studies showed that deletion of RAGE provides protection from the lethal effects of septic shock and the application of soluble RAGE, an extracellular decoy for RAGE ligands, improves survival in mice in sepsis [13,14,15]. Recent evidence revealed that RAGE mediated LPS-induced inflammation in alveolar type I epithelial cells via activating NF-κB [16]. Moreover, RAGE/NF-κB signaling pathway also played an important role in LPS-triggered inflammatory lung injury [17]. Previous reports also showed that AGEs, tumor necrosis factor-alpha (TNF-alpha), and 17 beta-estradiol significantly up-regulated RAGE mRNA and protein levels through NF-κB in human microvascular endothelial cells [18]. However, the effects of LPS on RAGE expression and the role of RAGE in LPS-induced activation in HUVECs are not well illustrated.

We have previously demonstrated that S100A9-induced monolayer hyperpermeability in human umbilical vein endothelial cells (HUVECs) was dependent on RAGE [19]. Mounting evidence implied that HMGB1-induced endothelial hyperpermeability is also mediated via RAGE [20,21]. We have confirmed that RAGE was critically involved in rearrangement of cytoskeletal filament F-actin caused by LPS [22]. Therefore, we wonder whether RAGE has a role in LPS-mediated endothelial barrier dysfunction.

The aim of the present study was to further investigate the effects of RAGE on LPS-induced NF-κB activation and endothelial barrier disruption.

## 2. Materials and Methods

### 2.1. Reagents

HUVECs were purchased from ScienCell (Carlsbad, CA, USA). DMEM/F12 medium, fetal bovine serum (FBS), trypsin, glutamine, penicillin, and streptomycin were all from Gibco BRL (Grand Island, NY, USA). LPS from *Escherichia coli* 055:B5 was obtained from Sigma (St. Louis, MO, USA). Antibodies against p-IκBα and IκBα were from Santa Cruz Biotechnology (Santa Cruz, CA, USA). Antibodies recognizing NF-κB p65 and β-actin were from Cell Signaling (Beverly, MA, USA). Secondary antibody was from Biosynthesis (Beijing, China). Human RAGE blocking antibody was obtained from R&D systems (Minneapolis, MN, USA) and at 10 μg/mL, this antibody will block 90% of RAGE binding. This antibody was used as primary antibody for western blotting as well. Unless specified, biochemical reagents were obtained from Sigma (St. Louis, MO, USA).

### 2.2. Cell Culture

HUVECs were cultured in DMEM/F12 containing 10% FBS at 37 °C in a humidified atmosphere with 5% CO_2_. In all experiments, HUVECs were grown to 90% confluence and starved of serum for 12 h before being stimulated with LPS. In some experiments, HUVECs were pretreated with the RAGE blocking antibody for 60 min, followed by stimulation with LPS.

### 2.3. Western Blotting

HUVECs were harvested and lysed with ice-cold lysis buffer (20 mmol/L Tris pH 7.4, 2.5 mmol/L EDTA, 1% Triton X-100, 1% deoxycholic acid, 0.1% SDS, 100 mmol/L NaCl, 10 mmol/L NaF and 1 mmol/L Na_3_VO_4_) supplemented with protease and phosphatase inhibitors. The protein samples were separated using 12% SDS-PAGE, and then transferred to PVDF membranes. After being blocked with 5% bovine serum albumin (BSA), the membranes were incubated with primary antibodies directly against RAGE (1:200), p-IκBα (1:200), IκBα (1:200) and β-actin (1:1000) overnight at 4 °C, followed by incubation with a horseradish peroxidase-conjugated secondary antibody specific to the primary antibody for 1 h. After further washed, the membranes were treated with chemiluminescence reagents and the signals were imaged with an imaging station. Image J was used to measure the density of the bands.

### 2.4. Immunofluorescent Staining

To visually identify the translocation of NF-κB p65, HUVECs were plated on gelatin-coated glass-bottom microwell plates (Corning Costar, Corning, NY, USA) and grown to confluence. After LPS treatment, the cells were fixed and permeabilized with 3.7% formaldehyde and 0.5% Triton X-100 at room temperature. Then cells were washed twice with PBS, blocked with 5% BSA for 1 h at 37 °C, and incubated with NF-κB antibody (1:50) overnight at 4 °C. After a thorough wash with PBS, the cells were stained with an FITC-conjugated secondary antibody (1:200) against the primary antibody applied and nuclei were stained with 4’,6-diamidino-2-phenylindole (DAPI). The staining results were imaged using a Zeiss LSM780 laser confocal scanning microscope (Zeiss, Oberkochen, Germany).

### 2.5. Transendothelial Electrical Resistance (TER)

Transendothelial electrical resistance (TER) of HUVEC monolayer was determined using STX2 electrode and EVOM^2^ meter according to the instruction manual of manufacture (World Precision Instruments, Sarasota, FL, USA) [23]. Briefly, HUVECs were seeded at 0.5 × 10^5^/well in gelatin-coated, 6.5 mm transwell filters (0.4 mm pore size) and grown to confluence. Resistance values of multiple transwell inserts of an experimental group were measured sequentially and the mean was expressed in the common unit (Ωcm^2^) after subtraction of the value of a blank cell-free filter.

### 2.6. Endothelial Monolayer Permeability Assay

HUVECs were grown to confluence on transwell membranes and the tracer FITC-labeled dextran (1 mg/mL) was then added to the upper chambers for 45 min. Samples were collected from both the upper and lower chambers. Then the concentrations of dextran were determined with a HTS 7000 microplate reader. The permeability of endothelial monolayer were evaluated by the permeability coefficient of dextran calculated as follows: Pd = [A]/t × 1/A × V/[L], where [A] is the dextran concentration in bottom chamber, t refers to time in seconds, A indicates the area of the membrane (in cm^2^), V is the volume of the bottom chamber and [L] is the dextran concentration in upper chamber.

### 2.7. Statistical Analysis

All data were expressed as means ± s.d. from more than three independent experiments and analyzed using SPSS 16.0 software. Statistical comparisons were performed using one-way ANOVA and *p* < 0.05 was considered significant.

## 3. Results

### 3.1. LPS Upregulates the Protein Expression of RAGE in HUVECs

**Figure 1:**
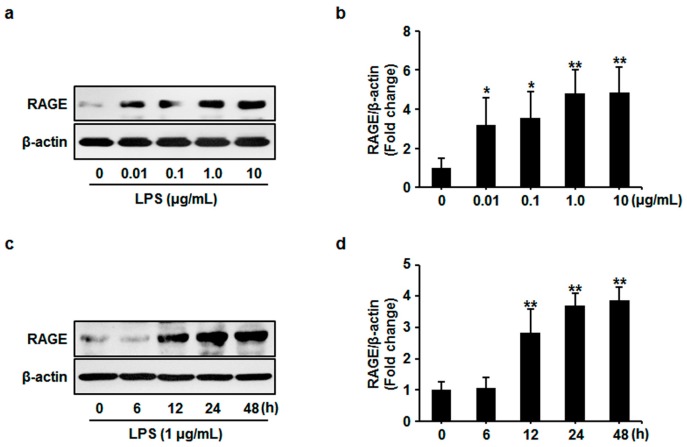
(**a**) Human umbilical vein endothelial cells (HUVECs) were treated with lipopolysaccharide (LPS) at the indicated dose for 24 h. Cell lysates were prepared and subjected to western blotting. Expression of receptor for advanced glycation end products (RAGE) was detected and representative images of three independent experiments were shown. (**b**) The ratio of RAGE immunopositivity to β-actin was calculated. The results are expressed as mean ± s.d. of three independent experiments. * *p* < 0.05 vs. untreated control, ** *p* < 0.01 vs. untreated control. (**c**) HUVECs were treated with LPS (1 μg/mL) for 0, 6, 12, 24 and 48 h respectively. Cell lysates were prepared and subjected to western blotting. Expression of RAGE was detected and representative images of three independent experiments were shown. (**d**) The ratio of *RAGE* immunopositivity to β-actin was calculated. The results are expressed as mean ± s.d. of three independent experiments. ** *p* < 0.05 vs. untreated control, ** *p* < 0.01 vs. untreated control.

**Figure 2:**
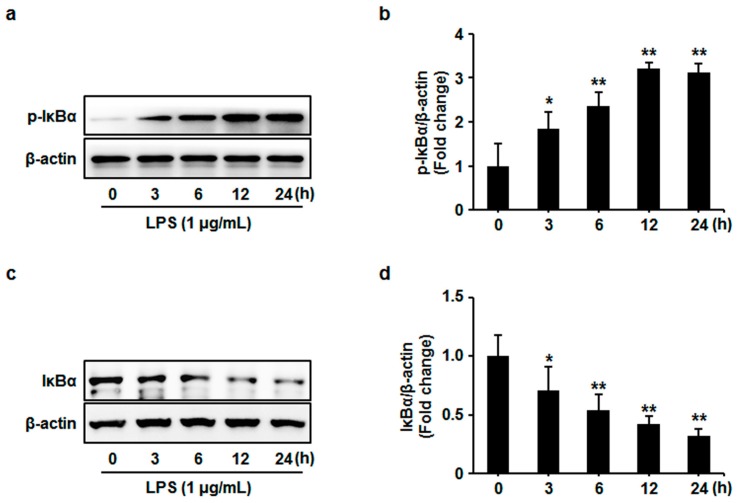
HUVECs were stimulated with LPS (1 μg/mL) for the indicated time. (**a**) Cell lysates were prepared and subjected to western blotting. Phosphorylation (*p*) of IκBα was examined and representative images of three independent experiments are shown. (**b**) The ratio of phosphorylated IκBα immunopositivity to β-actin was calculated. The results are expressed as mean ± s.d. of three independent experiments. ** *p* < 0.05 vs. untreated control, ** *p* < 0.01 vs. untreated control. (**c**) Cell lysates were prepared and subjected to western blotting. Expression of IκBα was examined and representative images of three independent experiments are shown. (**d**) The ratio of IκBα immunopositivity to β-actin was calculated. The results are expressed as mean ± s.d. of three independent experiments. * *p* < 0.05 vs. untreated control, ** *p* < 0.01 vs. untreated control.

Firstly, we investigated the effects of LPS on protein expression of RAGE. HUVECs were exposed to LPS (0, 0.01, 0.1, 1 and 10 μg/mL) for 24 h. The significant increase of RAGE protein expression was observed in cells subjected to LPS at 0.01 μg/mL, which became more significant at 1 and 10 μg/mL (Figure 1a,b). Accordingly, 1 μg/mL LPS was applied for the following experiments. In the time course study, we found that RAGE expression significantly increased to a prolonged plateau level from 12 to 48 h after LPS application (Figure 2a,b). These results indicate that LPS upregulates the protein expression of RAGE in HUVECs, which may be involved in the pathogenesis of LPS-evoked endothelium dysfunction.

### 3.2. The Blockade of RAGE Inhibits LPS-Induced Phosphorylation and Degradation of IκBα

At first, the influences of LPS on the phosphorylation and subsequent degradation of IκBα were determined by western blotting. HUVECs were treated with LPS (1 μg/mL) for 0, 3, 6, 12 and 24 h. Then, the phosphorylated and total IκBα were examined. We found that LPS significantly increased the phosphorylation of IκBαin a time-dependent manner. IκBα phosphorylation significantly increased at 3 h, gradually reached a maximum at 12 h, and then slightly decreased from 12 to 24 h after LPS treatment (Figure 2a,b). LPS also significantly decreased the expression of IκBα from 3 to 24 h in HUVECs (Figure 2c,d). These results were consistent with the previous report that LPS contributed to phosphorylation and degradation of IκBα, and thereby activated NF-κB [24].

**Figure 3:**
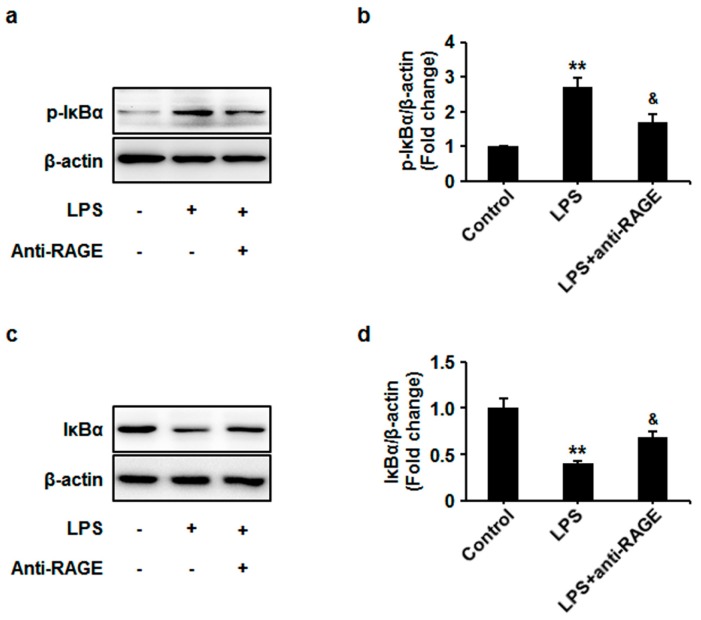
HUVECs were pretreated with anti-RAGE (10 μg/mL) for 1 h and followed by LPS (1 μg/mL) stimulation for 12 h. (**a**) Cell lysates were prepared and subjected to western blotting. Phosphorylation (*p*) of IκBα was examined and representative images of three independent experiments are shown. (**b**) The ratio of phosphorylated IκBα immunopositivity to β-actin was calculated. The results are expressed as mean ± s.d. of three independent experiments. ** *p* < 0.01 vs. untreated control, ^&^ *p* < 0.05 vs. LPS; (**c**) Cell lysates were prepared and subjected to western blotting. Expression of IκBα was examined and representative images of three independent experiments are shown. (**d**) The ratio of IκBα immunopositivity to β-actin was calculated. The results are expressed as mean ± s.d. of three independent experiments. ** *p* < 0.01 vs. untreated control, ^&^ *p* < 0.05 vs. LPS.

Furthermore, to evaluate the role of RAGE in LPS-induced phosphorylation and degradation of IκBα, the RAGE blocking antibody was used before LPS treatment in HUVECs. The blockade of RAGE significantly inhibited LPS-induced IκBα phosphorylation and degradation (Figure 3). These results suggest that RAGE may be involved in LPS-triggered NF-κB activation.

### 3.3. LPS-Induced NF-κB Nuclear Translocation is Attenuated by Blockade of RAGE

**Figure 4:**
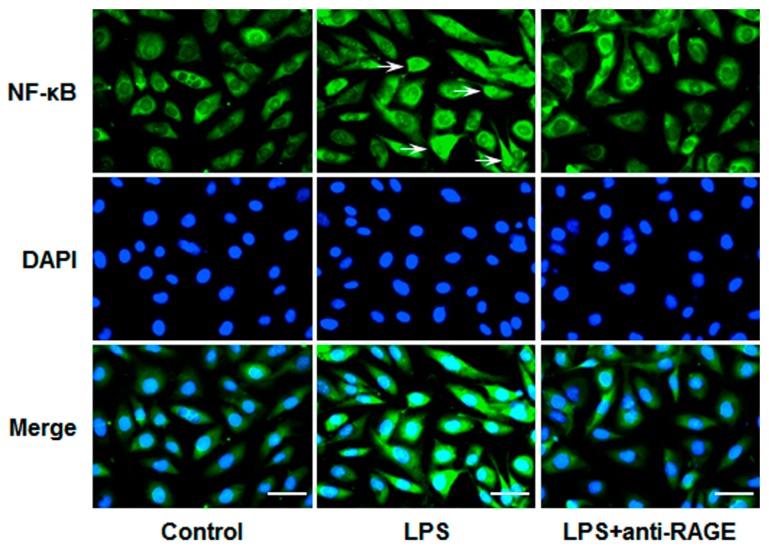
HUVECs grown on Petri dishes were pretreated with anti-RAGE (10 μg/mL) for 1 h and followed by LPS (1 μg/mL) stimulation for 12 h. Then cells were processed for immunostaining with anti-NF-κB p65 antibody. Nuclei of cells were stained with DAPI (blue) and p65 was visualized by green fluorescence. The arrows show the nuclear localization of NF-κB. Scale bars, 50 μm. Results are representative images of three independent experiments.

To confirm the role of RAGE in LPS-induced NF-κB activation, we then detected the distribution of NF-κB p65 in HUVECs by immunological fluorescent staining. The results showed that LPS significantly induced translocation of NF-κB p65 from cytoplasm to nuclear. However, the nuclear translocation of NF-κB p65 caused by LPS was remarkably attenuated by the RAGE blocking antibody (Figure 4). These data indicate that LPS-induced NF-κB activation is, at least partially, mediated by RAGE.

### 3.4. The Blockade of RAGE Diminishes LPS-Induced Endothelial Hyperpermeability

**Figure 5:**
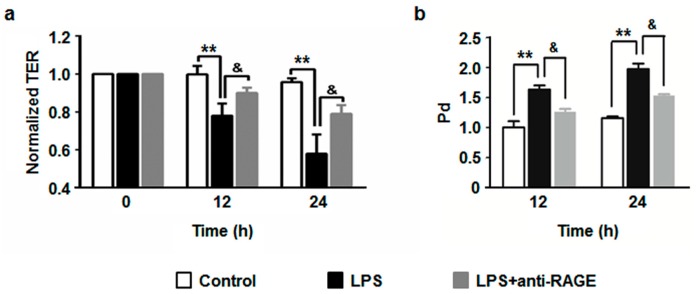
HUVECs were pretreated with anti-RAGE (10 μg/mL) for 1 h or not, and then followed by LPS (1 μg/mL) stimulation for 24 h. (**a**) The TER was measured at 0, 12 and 24 h respectively. (**b**) The Pd value was measured at 12 and 24 h as described in Methods. The results are expressed as mean ± s.d. of three independent experiments. ** *p* < 0.01 vs. untreated control, ^&^ *p* < 0.01 vs. LPS.

Our previous report showed that LPS significantly decreased transendothelial electrical resistance (TER) in HUVECs [9]. In the current experiment, we observed a longer action of LPS on endothelial barrier dysfunction. HUVECs were treated with LPS (1 μg/mL) for 0, 12 and 24 h, respectively. The TER value remarkably dropped at 12 h, and the level fell lower at 24 h (Figure 5a). These results suggest that LPS contribute to a long-lasting compromise of endothelial barrier function. In order to clarify the role of RAGE in LPS-induced endothelial hyperpermeability, HUVECs were pretreated with the RAGE blocking antibody for 1 h followed by stimulation with LPS. The results showed that the RAGE blocking antibody partially abrogated the decrease of TER (Figure 5a). To further confirm the role of RAGE in LPS-induced endothelial hyperpermeability, we performed an experiment by measuring the leakage of FITC-labeled dextran through endothelial monolayer. In accordance with TER changes, the results showed that LPS elicited a remarkable increase in FITC-dextran leakage, while pretreatment with RAGE blocking antibody significantly ameliorated this effect (Figure 5b). As a whole, these results indicate that RAGE is crucially involved in LPS-mediated endothelial barrier dysfunction.

## 4. Discussion

Sepsis is characterized by hyperactivation of the inflammatory system. Despite intensive studies over decades, few new drugs against sepsis have been developed. With a hospital mortality rate greater than 40%, sepsis remains a tremendous challenge now [25]. Administration of LPS to HUVECs is a well-established cell model to study the mechanism of vascular inflammation and endothelial barrier disruption in sepsis. Our findings may provide some clues about potential intervention strategies for sepsis.

Extracellular signal transduction into the cell in response to LPS remains elusive. Toll-like receptor 4 (TLR4) is the central signaling receptor for LPS in mammals [26]. Several molecules such as LPS binding protein (LBP), CD14 and MD2 are required for the recognition of LPS by TLR4. Afterwards, RAGE was found to be a pattern recognition receptor that can bind to LPS as well. Previous studies showed that RAGE can associate with LPS and regulate inflammatory responses during septic shock [27].

Importantly, significant correlation between NF-κB activity and concentrations of pro-inflammatory mediators was revealed in patients with sepsis [28]. The activation of NF-κB leads to production of pro-inflammatory molecules. Although numerous studies reported that RAGE played an important role in NF-κB activation in diverse cell types, few reports were concerned with LPS-treated endothelial cells. Therefore, we observed the role of RAGE in LPS-induced NF-κB activation in HUVECs. Our results showed that LPS significantly increased IκBα phosphorylation and degradation, while blockade of RAGE significantly abolished these effects. To further evaluate the effects of RAGE on NF-κB activation, the translocation of NF-κB p65 was detected with immunofluorescence staining. The data showed that the RAGE blocking antibody significantly inhibited LPS-induced NF-κB p65 nuclear translocation in HUVECs. Collectively, our results indicate that LPS can activate NF-κB through RAGE in HUVECs, though it is a classic pathway that LPS activates NF-κB through TLR4. Previous studies reported that HMGB1 induced the crosstalk between TLR4 and RAGE [29]. However, it is unknown whether TLR4 and RAGE cooperate with each other in response to LPS. Moving forward, further studies are needed to be done in terms of this signaling axis.

Notably, one feature of RAGE-mediated NF-κB activation is the sustained effect in time course. Therefore, prolonged activation of NF-κB enhances the expression of RAGE, forming a positive feedback loop [21,30]. We observed an LPS-induced dose- and time-dependent increase of RAGE expression. Moreover, increased RAGE expression seemed to provide more receptors for LPS to bind with and support a prolonged and stronger activation of NF-κB. In turn, the elevation of RAGE protein level was probably a consequence of NF-κB activation. Previous studies showed that inhibition of NF-κB activation significantly ameliorated LPS-induced increase of RAGE expression [16,17]. As shown in our results, a notable increase of IκBα phosphorylation and subsequent IκBα degradation appeared as early as 3 h after LPS application, but the elevated RAGE expression was not observed until 12 h after LPS treatment. Additionally, blockade of RAGE significantly ameliorated LPS-induced NF-κB activation. Taken together, it is most likely that this positive feedback loop functions well under LPS treatment.

The clinical manifestations of progressive edema occurring in tissues and organs of septic patients suggest an elevated vascular permeability. Although there is uncertainty about whether the result of vascular dysfunction is due to the direct effects of LPS on the endothelium or is secondary to the release of inflammatory mediators. A recombinant endotoxin-neutralizing protein blocked LPS-induced endothelial hyperpermeability [31], indicating that LPS itself can lead to the disruption of vascular barrier. Afterwards, we reported that LPS could induce actin cytoskeleton alteration and lead to intercellular gap formation [32]. Moreover, we demonstrated that LPS significantly induced endothelial monolayer hyperpermeability response [33]. Yet, the mechanisms involved in LPS-induced endothelial hyperpermeability were not fully understood.

We previously found that the RAGE blocking antibody attenuated AGE-induced elevation in endothelial permeability [34]. RAGE knockout by siRNA transfection inhibited AGE-exerted TER decrease and FITC-dextran leakage in HUVECs (data not published). In contrary, overexpression of RAGE amplified AGE-mediated endothelial hyperpermeability (data not published). We have revealed a significant exudation of FITC-dextran from mesenteric venules in AGE-treated wild type mice. However, this exudation was markedly reduced in RAGE knockout mice, which further confirmed the role of RAGE in vascular hyperpermeability in vivo [35]. Moreover, we have shown that LPS caused significant rearrangement of cytoskeletal filament F-actin in insolated wild-type mouse pulmonary microvascular endothelial cells (PMVECs). However, no such changes were detected in PMVECs isolated from RAGE gene-knockout mice [22]. Our previous studies showed that the binding of RAGE with its ligands contributed to an increased vascular permeability and RAGE was critically involved in LPS-induced rearrangement of cytoskeletal filament F-actin in endothelial cells [19,22,34,35]. Therefore, we postulate that RAGE may also be implicated in LPS-triggered endothelial hyperpermeability. Thus, we performed TER assay and endothelial monolayer permeability assay to examine this hypothesis. Consistently, LPS elicited a dramatic decrease of TER value, as well as an increase of dextran leakage, indicating a disruption endothelial barrier. However, blockade of RAGE significantly attenuated LPS-induced endothelial hyperpermeability. These results suggest that RAGE play an important role in LPS-induced endothelial barrier dysfunction.

In line with most previous observations, He et al. reported that NF-κB activation was critically involved in LPS-induced tight junction disruption and barrier dysfunction in brain microvascular endothelial cells [36]. In addition, endothelial barrier maintaining protein caveolin1 was also involved in endothelial hyperpermeability [37]. A significant increase of caveolin1 expression was achieved after LPS stimulation [38]. Moreover, inhibition of NF-κB activation inhibited LPS-induced caveolin1 expression and endothelial hyperpermeability [38].

Adherens junction proteins such as VE-cadherin and β-catenin play important roles in endothelial hyperpermeability. Phosphorylation of VE-cadherin has been implicated in the increase of endothelial permeability. Previous studies showed that LPS induced NF-κB activation and VE-cadherin phosphorylation [39]. We tentatively put forward that LPS induces VE-cadherin phosphorylation, which probably leads to the internalization of VE-cadherin and a disruption of adherens junction. However, whether this process is dependent on RAGE-mediated NF-κB activation needs to be clarified. As is known, β-catenin is constitutively bound to VE-cadherin in an inactive state. Upon inflammatory stimulation, the cadherin–catenin complex may be disrupted. Then, β-catenin was released and stabilized before translocated to nucleus and modulate gene transcription [40]. We hypothesize that LPS may cause the disruption of cadherin–catenin complex and thus induce the translocation of β-catenin into nucleus, which probably contributes to the transcription of permeability-increasing proteins. Nevertheless, the β-catenin signaling networks are quite elusive, for free β-catenin was able to physically complex with NF-κB [41]. Our future studies will explore the relationships among these agents in the pathogenesis of endothelial barrier disruption.

## 5. Conclusions

In summary, the results of our present study indicate that RAGE is involved in LPS-induced NF-κB activation and endothelial hyperpermeability. And these results may provide new diagnostic and therapeutic options in patients with sepsis.

## References

[1] Opal S.M., Laterre P.F., Francois B., LaRosa S.P., Angus D.C., Mira J.P., Wittebole X., Dugernier T., Perrotin D., Tidswell M. (2013). Effect of eritoran, an antagonist of MD2-TLR4, on mortality in patients with severe sepsis: The ACCESS randomized trial. JAMA.

[2] Ranieri V.M., Thompson B.T., Barie P.S., Dhainaut J.F., Douglas I.S., Finfer S., Gardlund B., Marshall J.C., Rhodes A., Artigas A., Payen D. (2012). Drotrecogin alfa (activated) in adults with septic shock. N. Engl. J. Med..

[3] Abraham E. (2003). Nuclear factor-κB and its role in sepsis-associated organ failure. J. Infect. Dis..

[4] Ghosh S., Hayden M.S. (2008). New regulators of NF-κB in inflammation. Nat. Rev. Immunol..

[5] Baeuerle P.A., Baltimore D. (1988). I κB: A specific inhibitor of the NF-κB transcription factor. Science.

[6] Yoo J.W., Moon J.Y., Hong S.B., Lim C.M., Koh Y., Huh J.W. (2015). Clinical significance of circulating endothelial cells in patients with severe sepsis or septic shock. Infect. Dis..

[7] Ye X., Ding J., Zhou X., Chen G., Liu S.F. (2008). Divergent roles of endothelial NF-κB in multiple organ injury and bacterial clearance in mouse models of sepsis. J. Exp. Med..

[8] Groger M., Pasteiner W., Ignatyev G., Matt U., Knapp S., Atrasheuskaya A., Bukin E., Friedl P., Zinkl D., Hofer-Warbinek R. (2009). Peptide Bbeta (15–42) preserves endothelial barrier function in shock. PLoS ONE.

[9] Xia J.L., Wang L.Q., Wu L.L., Huang Q.B. (2014). Doxycycline hyclate protects lipopolysaccharide-induced endothelial barrier dysfunction by inhibiting the activation of p38 mitogen-activated protein kinase. Biol. Pharm. Bull..

[10] Schmidt A.M., Vianna M., Gerlach M., Brett J., Ryan J., Kao J., Esposito C., Hegarty H., Hurley W., Clauss M. (1992). Isolation and characterization of two binding proteins for advanced glycosylation end products from bovine lung which are present on the endothelial cell surface. J. Biol. Chem..

[11] Hofmann M.A., Drury S., Fu C., Qu W., Taguchi A., Lu Y., Avila C., Kambham N., Bierhaus A., Nawroth P. (1999). RAGE mediates a novel proinflammatory axis: A central cell surface receptor for S100/calgranulin polypeptides. Cell.

[12] Hori O., Brett J., Slattery T., Cao R., Zhang J., Chen J.X., Nagashima M., Lundh E.R., Vijay S., Nitecki D. (1995). The receptor for advanced glycation end products (RAGE) is a cellular binding site for amphoterin. Mediation of neurite outgrowth and co-expression of rage and amphoterin in the developing nervous system. J. Biol. Chem..

[13] Bopp C., Bierhaus A., Hofer S., Bouchon A., Nawroth P.P., Martin E., Weigand M.A. (2008). Bench-to-bedside review: The inflammation-perpetuating pattern-recognition receptor RAGE as a therapeutic target in sepsis. Crit. Care.

[14] Christaki E., Lazaridis N., Opal S.M. (2012). Receptor for advanced glycation end products in bacterial infection: Is there a role for immune modulation of receptor for advanced glycation end products in the treatment of sepsis?. Curr. Opin. Infect. Dis..

[15] Liliensiek B., Weigand M.A., Bierhaus A., Nicklas W., Kasper M., Hofer S., Plachky J., Grone H.J., Kurschus F.C., Schmidt A.M. (2004). Receptor for advanced glycation end products (RAGE) regulates sepsis but not the adaptive immune response. J. Clin. Investig..

[16] Li Y., Wu R., Zhao S., Cheng H., Ji P., Yu M., Tian Z. (2014). RAGE/NF-κB pathway mediates lipopolysaccharide-induced inflammation in alveolar type I epithelial cells isolated from neonate rats. Inflammation.

[17] Li Y., Wu R., Tian Y., Yu M., Tang Y., Cheng H., Tian Z. (2015). RAGE/NF-κB signaling mediates lipopolysaccharide induced acute lung injury in neonate rat model. Int. J. Clin. Exp. Med..

[18] Tanaka N., Yonekura H., Yamagishi S., Fujimori H., Yamamoto Y., Yamamoto H. (2000). The receptor for advanced glycation end products is induced by the glycation products themselves and tumor necrosis factor-alpha through nuclear factor-κB, and by 17beta-estradiol through Sp-1 in human vascular endothelial cells. J. Biol. Chem..

[19] Wang L., Luo H., Chen X., Jiang Y., Huang Q. (2014). Functional characterization of S100A8 and S100A9 in altering monolayer permeability of human umbilical endothelial cells. PLoS ONE.

[20] Huang W., Liu Y., Li L., Zhang R., Liu W., Wu J., Mao E., Tang Y. (2012). HMGB1 increases permeability of the endothelial cell monolayer via RAGE and Src family tyrosine kinase pathways. Inflammation.

[21] Wolfson R.K., Chiang E.T., Garcia J.G. (2011). HMGB1 induces human lung endothelial cell cytoskeletal rearrangement and barrier disruption. Microvasc. Res..

[22] Zhou X.Y., Zhang W.J., Huang Q.B., Guo X.H. (2015). Role of RAGE in lipopolysaccharide-induced cytoskeletal changes in mouse pulmonary microvascular endothelial cells. J. South Med. Univ..

[23] Patabendige A., Skinner R.A., Abbott N.J. (2013). Establishment of a simplified in vitro porcine blood-brain barrier model with high transendothelial electrical resistance. Brain Res..

[24] Xing J., Birukova A.A. (2010). ANP attenuates inflammatory signaling and Rho pathway of lung endothelial permeability induced by LPS and TNFalpha. Microvasc. Res..

[25] Singer M., Deutschman C.S., Seymour C.W., Shankar-Hari M., Annane D., Bauer M., Bellomo R., Bernard G.R., Chiche J.D., Coopersmith C.M. (2016). The Third International Consensus Definitions for Sepsis and Septic Shock (Sepsis-3). JAMA.

[26] Poltorak A., He X., Smirnova I., Liu M.Y., Van Huffel C., Du X., Birdwell D., Alejos E., Silva M., Galanos C. (1998). Defective LPS signaling in C3H/HeJ and C57BL/10ScCr mice: Mutations in Tlr4 gene. Sience.

[27] Yamamoto Y., Harashima A., Saito H., Tsuneyama K., Munesue S., Motoyoshi S., Han D., Watanabe T., Asano M., Takasawa S. (2011). Septic shock is associated with receptor for advanced glycation end products ligation of LPS. J. Immunol..

[28] Arnalich F., Garcia-Palomero E., Lopez J., Jimenez M., Madero R., Renart J., Vazquez J.J., Montiel C. (2000). Predictive value of nuclear factor κB activity and plasma cytokine levels in patients with sepsis. Infect. Immun..

[29] Qin Y.H., Dai S.M., Tang G.S., Zhang J., Ren D., Wang Z.W., Shen Q. (2009). HMGB1 enhances the proinflammatory activity of lipopolysaccharide by promoting the phosphorylation of MAPK p38 through receptor for advanced glycation end products. J. Immunol..

[30] Bierhaus A., Humpert P.M., Morcos M., Wendt T., Chavakis T., Arnold B., Stern D.M., Nawroth P.P. (2005). Understanding RAGE, the receptor for advanced glycation end products. J. Mol. Med..

[31] Bannerman D.D., Fitzpatrick M.J., Anderson D.Y., Bhattacharjee A.K., Novitsky T.J., Hasday J.D., Cross A.S., Goldblum S.E. (1998). Endotoxin-neutralizing protein protects against endotoxin-induced endothelial barrier dysfunction. Infect. Immun..

[32] Huang Q.B., Song L., Zhao K.S., Chen B., Huang X.L. (2004). Effects of lipopolysaccharide on actin reorganization and actin pools in endothelial cells. Chin. J. Traumatol..

[33] Du J., Zeng C., Li Q., Chen B., Liu H., Huang X., Huang Q. (2012). LPS and TNF-alpha induce expression of sphingosine-1-phosphate receptor-2 in human microvascular endothelial cells. Pathol. Res. Pract..

[34] Guo X., Wang L., Chen B., Li Q., Wang J., Zhao M., Wu W., Zhu P., Huang X., Huang Q. (2009). ERM protein moesin is phosphorylated by advanced glycation end products and modulates endothelial permeability. Am. J. Physiol. Heart Circ. Physiol..

[35] Zhang W., Xu Q., Wu J., Zhou X., Weng J., Xu J., Wang W., Huang Q., Guo X. (2015). Role of Src in Vascular Hyperpermeability Induced by Advanced Glycation End Products. Sci. Rep..

[36] He F., Peng J., Deng X.L., Yang L.F., Wu L.W., Zhang C.L., Yin F. (2011). RhoA and NF-κB are involved in lipopolysaccharide-induced brain microvascular cell line hyperpermeability. Neuroscience.

[37] Schlegel N., Leweke R., Meir M., Germer C.T., Waschke J. (2012). Role of NF-κB activation in LPS-induced endothelial barrier breakdown. Histochem. Cell Biol..

[38] Tiruppathi C., Shimizu J., Miyawaki-Shimizu K., Vogel S.M., Bair A.M., Minshall R.D., Predescu D., Malik A.B. (2008). Role of NF-κB-dependent caveolin-1 expression in the mechanism of increased endothelial permeability induced by lipopolysaccharide. J. Biol. Chem..

[39] Zhang Y., Sun K., Liu Y.Y., Zhang Y.P., Hu B.H., Chang X., Yan L., Pan C.S., Li Q., Fan J.Y. (2014). Ginsenoside Rb1 ameliorates lipopolysaccharide-induced albumin leakage from rat mesenteric venules by intervening in both trans- and paracellular pathway. Am. J. Physiol.-Gastrointest. Liver Physiol..

[40] Nusse R. (2005). Wnt signaling in disease and in development. Cell Res..

[41] Deng J., Miller S.A., Wang H.Y., Xia W., Wen Y., Zhou B.P., Li Y., Lin S.Y., Hung M.C. (2002). β-catenin interacts with and inhibits NF-κB in human colon and breast cancer. Cancer Cell.

